# Recurrent Midgut Bleeding due to Jejunal Angioleiomyoma

**DOI:** 10.1155/2016/4569781

**Published:** 2016-09-07

**Authors:** Mahir Gachabayov, Petr Mityushin

**Affiliations:** Department of Abdominal Surgery, Vladimir City Clinical Hospital of Emergency Medicine, Gorky Street 5, Vladimir 600017, Russia

## Abstract

Angioleiomyoma being a type of true smooth muscle gastrointestinal tumors can lead to serious life-threatening gastrointestinal bleeding. We report a case of 21-year-old male patient with recurrent midgut bleeding. Contrast-enhanced CT revealed highly vascular small bowel neoplasm. The patient underwent laparotomy with bowel resection and recovered uneventfully. Histopathology revealed jejunal angioleiomyoma.

## 1. Introduction

Obscure gastrointestinal bleeding (GIB) is persistent or recurrent bleeding from the gastrointestinal (GI) tract after negative evaluations with upper and lower endoscopies accounting for 5% of all GIB cases [1, 2]. Midgut GIB makes up to 80% of all obscure GIB cases [3]. Small bowel tumors are responsible for 10–20% of these cases of midgut GIB in Western countries [1]. Angioleiomyomata of small bowel, especially those complicated by GIB, are very rare.

## 2. Case Report

A 21-year-old male student from another city but studying in Vladimir was admitted to Vladimir City Clinical Hospital of Emergency Medicine with a 2-day history of melena and fatigue. His past medical history was significant for peptic ulcer disease because of which he was exempted from military service, recurrent epistaxis due to septal deviation, and chronic iron-deficiency anemia. On admission his skin was pale, heart rate 98 bpm, blood pressure 110/60 mmHg, and hemoglobin 6.2 mg/dL. On EGD a flat duodenal ulcer (0.8 cm with fibrin-covered base) was revealed. PPIs and packed RBCs (2 doses) started immediately and after 3 days the patient was transferred to the department of internal medicine with Hb of 8.4 mg/dL and BP of 120/70 mmHg.

Eight days after discharge from our department the patient was admitted again with recurrent melena and fatigue during 10 hours. On admission, HR was 104 bpm, BP 80/60 mmHg, and Hb 5.5 mg/dL. Healthily discharged patient came back with the fear of dying. EGD was unremarkable. After IV fluids with 2 doses of packed RBCs and PPIs the patient was prepared for a colonoscopy with laxatives. Colonoscopy also appeared to be unremarkable. Then the patient was consulted by otolaryngologists to rule out possible posterior epistaxis. Endoscopic rhinoscopy revealed eroded nasal polyp without signs of ongoing bleeding which was excised. The patient was discharged after 2 days with BP 110/70 mmHg and Hb 7.7 mg/dL.

**Figure 1:**
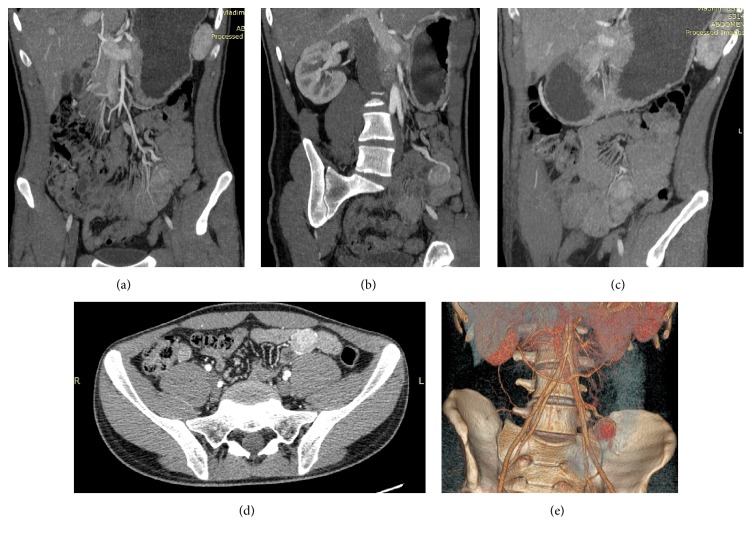
Contrast-enhanced CT showing highly vascular small bowel neoplasm: (a) coronal section, (b) and (c) oblique sections, (d) axial section, and (e) 3D reconstruction.

**Figure 2:**
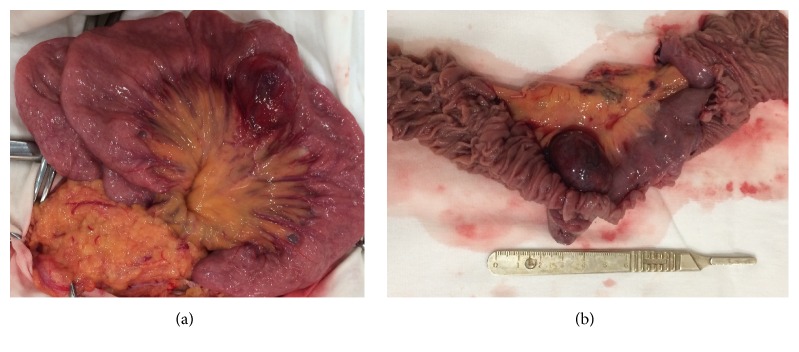
(a) Intraoperative and (b) postresection macroscopic appearance of small bowel angioleiomyoma.

**Figure 3:**
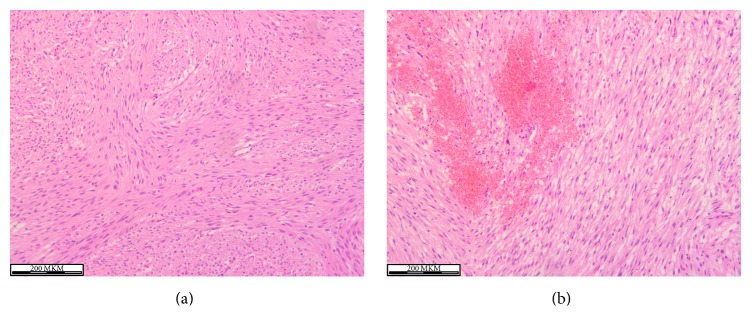
Histopathology of angioleiomyoma showing (a) intersecting bundles of spindle cells and (b) capillary channels with blood elements.

Seven days after discharge the patient was admitted again with 6-hour severe GI bleeding with BP 70/50 mmHg and Hb 4.9 mg/dL when we realized that we dealt with obscure midgut GI bleeding. After stabilization by infusing IV crystalloids and colloids, 4 doses of packed RBCs, and 4 doses of fresh frozen plasma the patient underwent contrast-enhanced multislice CT which revealed a highly vascular small bowel tumor (Figure 1). Considering high risk of recurrent bleeding urgent laparotomy and small bowel resection were performed which proved the diagnosis of jejunal neoplasm (Figure 2). Postoperatively, oral feeds resumed on the 2nd postoperative day, the wound stitches were taken off on 8th postoperative day, and the patient was discharged with BP 120/70 mmHg and Hb 9.8 mg/dL. At 3-month follow-up the patient was feeling well; his Hb was 15.4 mg/dL. The histopathology revealed bundles of spindle cells oriented perpendicularly to each other with bright eosinophilic cytoplasm and variety of small capillary channels with blood elements (Figure 3). The immunohistochemistry was positive for smooth muscle antigens and negative for GIST antigens.

## 3. Discussion

The most common location of leiomyoma is the uterus (95%), followed by skin (3%) and GI tract (1.5%) [4]. True smooth muscle neoplasms (leiomyomata) are the second most common mesenchymal neoplasms in GI tract accounting for 32% [5]. The World Health Organization defined leiomyoma in 1969 to be a “circumscribed benign, often cutaneous tumor composed of intersecting bundles of mature smooth muscle cells” and classified it into three groups: solid leiomyoma, vascular leiomyoma (angioleiomyoma), and epithelioid leiomyoma (leiomyoblastoma) [6].

Proposed theories to describe the origin of vascular leiomyoma include progression from aberrant undifferentiated mesenchyme, progression from vascular malformation, and neoplastic proliferation of smooth muscles of the walls of the vasculature [7].

In 1973 Morimoto classified angioleiomyomata dividing them into three histopathologic subtypes: (1) capillary or solid: closely compacted smooth muscle with number of small, slit-like vascular channels (this type is the most common), (2) venous: vascular channels with thick, easily identifiable muscular walls, and (3) cavernous: the vascular channels dilated with less smooth muscle [8].

Clinical presentation of angioleiomyoma is varicolored and mostly correlated with complication. Uncomplicated angioleiomyomata are generally asymptomatic. Due to very small number of reported cases of this rare clinical entity it appears to be difficult to estimate clinical features and complication rate. Turan et al. analyzing 13 patients with complicated small bowel tumor found out that intestinal obstruction is the most common complication (7 out of 13 patients) followed by perforation (5 of 13 patients) [9].

To the best of our knowledge, 9 cases of gastrointestinal angioleiomyomata have been reported in medical literature until today. These cases are shown in Table 1 alongside our case. It seems that GIB is the most common complication of angioleiomyoma.

Analyzing 562 cases of angioleiomyoma of all locations Hachisuga et al. found preponderance in female with a ratio of 1.7 : 1 [10]. Comparing our case with previously reported cases with known data, our case appears to be the first case with male patient (Table 1). Moreover, our patient is the youngest. Most of the patients are older than 40 years.

In most previously reported cases radiology emerged to be more common. Contrast-enhanced CT appeared to be accurate in three cases including our case. Previous studies showed CT scan and scintigraphy to be sensitive for small bowel tumors [11, 12]. Takeshita et al. showed video capsule endoscopy and double-balloon enteroscopy to be beneficial for small bowel lesions [13]. Immunohistochemistry is crucial in the diagnosis of mesenchymal tumor and differentiation of malignant and suspicious high risk tumors [14]. The definitive treatment of angioleiomyoma is resection.

To conclude, small bowel angioleiomyoma is rare but life-threatening cause of midgut gastrointestinal bleeding. Contrast-enhanced tomography should be performed to a patient with obscure gastrointestinal bleeding after negative gastroscopy and colonoscopy.

## Figures and Tables

**Table 1:** 

Author	Age, gender	Location	Complication	Diagnosis	Treatment
Valnicek 1959 [15]	n/a	Small bowel	GIB	n/a	n/a
Gadaleanu and Popescu 1988 [16]	31, female	Duodenojejunal flexure	GIB	Laparotomy	2 stages: (1) tumor vascular pedicle ligation and (2) resection
Sapelkin 1989 [17]	n/a	Small bowel	Perforation	n/a	n/a
Pidoprigora et al. 1995 [18]	n/a	Small bowel	GIB	n/a	n/a
Sadat et al. 2007 [19]	58, female	Ileum	GIB	Angiography	Resection
Erdogan et al. 2007 [11]	64, female	Jejunum	Noncomplicated	Scintigraphy + CT (concurrent to colonic angiodysplasia)	Resection (+ subtotal colectomy)
Nakatani et al. 2010 [20]	45, female	Ileum	GIB	Capsule endoscopy + double-balloon enteroscopy	Resection
Turan et al. 2010 [9]	Age not known, female	Ileum	Intussusception	CT	Resection
Stanojević et al. 2013 [21]	40, female	Rectum	Prolapse	Clinical	Tumor excision
Our case	21, male	Jejunum	GIB	CT	Resection
